# Valproic acid suppresses collagen by selective regulation of Smads in conjunctival fibrosis

**DOI:** 10.1007/s00109-015-1358-z

**Published:** 2015-10-27

**Authors:** Li-Fong Seet, Li Zhen Toh, Sharon N. Finger, Stephanie W. L. Chu, Branko Stefanovic, Tina T. Wong

**Affiliations:** Ocular Therapeutics and Drug Delivery, Singapore Eye Research Institute, Singapore, Singapore; Department of Ophthalmology, Yong Loo Lin School of Medicine, National University of Singapore, Singapore, Singapore; Duke-NUS Graduate Medical School Singapore, Singapore, Singapore; Department of Biomedical Sciences, College of Medicine, Florida State University, Tallahassee, FL USA; Glaucoma Service, Singapore National Eye Center, 11 Third Hospital Avenue, Singapore, 168751 Singapore; School of Materials Science and Engineering, Nanyang Technological University, Singapore, Singapore

**Keywords:** Collagen, SMAD, TGF‐β2, Valproic acid, Fibrosis, Conjunctiva

## Abstract

**Abstract:**

Overproduction of type I collagen is associated with a wide range of fibrotic diseases as well as surgical failure such as in glaucoma filtration surgery (GFS). Its modulation is therefore of clinical importance. Valproic acid (VPA) is known to reduce collagen in a variety of tissues with unclear mechanism of action. In this report, we demonstrate that VPA inhibited collagen production in both conjunctival fibroblasts and the mouse model of GFS. In fibroblasts, VPA decreased type I collagen expression which intensified with longer drug exposure and suppressed steady-state type I collagen promoter activity. Moreover, VPA decreased Smad2, Smad3 and Smad4 but increased Smad6 expression with a similar intensity-exposure profile. Reduction of Smad3 using small hairpin RNA and/or overexpression of Smad6 resulted in decreased collagen expression which was exacerbated when VPA was simultaneously present. Furthermore, fibrogenic TGF-β2 failed to induce collagen when VPA was present, as opposed to the myofibroblast markers, beta-actin, alpha-smooth muscle actin and tenascin-C, which were elevated by TGF-β2. VPA suppressed p3TP-Lux luciferase activity and selectively rescued Smad6 expression from suppression by TGF-β2. Notably, SMAD6 overexpression reduced the effectiveness of TGF-β2 in inducing collagen expression. In corroboration, VPA inhibited type I collagen but increased Smad6 expression in the late phase of wound healing in the mouse model of GFS. Taken together, our data indicate that VPA has the capacity to effectively suppress both steady-state and fibrogenic activation of type I collagen expression by modulating Smad expression. Hence, VPA is potentially applicable as an anti-fibrotic therapeutic by targeting collagen.

**Key message:**

• VPA modulates type I collagen expression via members of the Smad family.

•VPA suppresses Smad2, Smad3 and Smad4 but upregulates Smad6.

•Smad3 and Smad6 are involved in VPA regulation of steady-state collagen expression.

•Smad6 is involved in VPA modulation of TGF-β-stimulated collagen expression.

•VPA reduces collagen and upregulates Smad6 in the mouse model of glaucoma filtration surgery.

## Introduction

Type I collagen is the major component of extracellular matrix of nearly every human organ and constitutes the most abundant form of collagen in the human body. The main physiological role of collagen is to provide an architectural scaffold to strengthen and support tissues. In response to injury or certain diseases, collagen production facilitates the restoration of tissue homeostasis and normal physiological functions [1]. At times, however, this tissue repair response may be exaggerated, resulting in fibrosis or scarring. Indeed, the excessive and abnormal accumulation of type I collagen is the hallmark of fibrosis, and evident in many fibroproliferative medical conditions where it may be life threatening when major organs such as the heart, lung and kidney are affected. Moreover, accumulation of type I collagen is prevalent in many tumours and associated with increased risk of metastasis and poor prognosis [2]. In some surgeries such as glaucoma filtration surgery (GFS), scarring is implicated to be the main cause of surgical failure [3]. Hence, the modulation of type I collagen production in these pathological contexts is of utmost biological and clinical importance.

TGF-β stimulation of collagen synthesis plays a key role in both physiological tissue repair and pathological fibrosis [1]. The core intracellular effectors of TGF-β signaling are Smads [4]. Interestingly, Smads are also known to regulate steady-state collagen expression [5]. Receptor-regulated Smads (R-Smads: Smad1, Smad2, Smad3, Smad5 and Smad8) are the downstream effectors of serine-threonine kinase receptors activated by binding to the TGF-β superfamily of ligands [6]. Generally, stimulation by fibrogenic TGF-β leads to phosphorylation of Smad2 and Smad3 while other ligands like the bone morphogenetic protein (BMP) induce phosphorylation of Smad1, Smad5 and Smad8. The common mediator Smad4 (Co-Smad) is not ligand-restricted and form heteromeric complexes with the activated R-Smads to accumulate in the nucleus where they are directly involved in the regulation of target gene transcription. On the other hand, inhibitory Smads (I-Smads: Smad6 and Smad7) act in opposition to the R-Smads to negatively regulate TGF-β signaling in a feedback loop.

Valproic acid (VPA) belongs to the short-chain fatty acid class of histone deacetylase (HDAC) inhibitors. VPA has been used for over two decades in the treatment of neurological disorders [7] and is currently under clinical investigation as an anticancer drug with promising outcomes [8]. Although HDAC has been suggested to be a potential target for fibrotic disorders [9], the effect of VPA on wound healing and fibrosis is obscure. VPA has been shown to reduce muscle collagen content [10] and renal fibrosis [11], inhibit collagen deposition and activation of hepatic stellate cells [12] as well as reduce cutaneous radiation syndrome in rats [13]. In apparent contradiction, VPA was reported to accelerate cutaneous wound healing, in part by increasing collagen production [14]. The molecular mechanism for VPA effects on wound healing is even less well understood. In renal tubular epithelial cells, VPA has been shown to reduce the mRNA expression of colony-stimulating factor-1 [15] or suppress epithelial-to-mesenchymal transition [16]. On the other hand, the reduction in the radiation-induced wound response due to VPA correlated with the suppression of aberrant TGF-β and tumour necrosis factor-α expression [13]. Clearly, the therapeutic potential of VPA to reduce collagen production and the associated mechanisms need further validation.

In this study, we investigated the effect and mechanism of VPA activity on type I collagen production in conjunctival fibrosis. Conjunctival fibroblasts have been implicated as the main effector cells that elicit the fibrotic response in GFS [17–19]. Our data revealed that VPA has the capacity to reduce type I collagen expression in conjunctival fibroblasts at the steady-state as well as in the presence of TGF-β2. Moreover, we determined that while the effect of VPA on basal collagen may involve several members of the Smad family, particularly Smad3 and Smad6, the inhibitory effect of VPA in the fibrogenic activation of collagen by TGF-β2 selectively involved Smad6. The in vitro findings were corroborated in the mouse model of GFS [20] where type I collagen and Smad6 were modulated by VPA in a similar manner. Taken together, our study indicates that VPA is an effective inhibitor of collagen production via the disruption of both steady-state and TGF-β regulatory pathways mediated in part by Smads.

## Materials and methods

### Primary conjunctival fibroblast cell culture, treatments and transfections

Primary conjunctival fibroblasts were obtained from the eyes of C57BL6/J mice and cultured as described previously [21]. Treatment with valproic acid (Sigma-Aldrich Co., St. Louis, MO), was carried out at 300 μg/ml (or 2 mM) for 72 h unless otherwise indicated. Treatment with TGF-β2 (PeproTech Inc., NJ, USA) was performed at 8 ng/ml. Transfections were performed using the P2 primary cell 4D Nucleofector kit L (Lonza, Basel, Switzerland) and the 4D-Nucleofactor X unit (Lonza) according to manufacturer’s protocol. The shRNA for mouse Smad3 (referred to as shSmad3 in the text) was a gift from Vicki Boussiotis (Addgene plasmid # 32806) while the negative control for this plasmid was the piGENETM U6 Rep vector purchased from iGene Therapeutics, Inc (Tsukuba, Japan). The expression vector for mouse Smad6 (referred to as pSmad6 in the text) was purchased from GE Healthcare Dharmacon Inc. (CO, USA) while the negative control for this plasmid was pcDNA3.1(-) (Invitrogen Corp., Carlsbad, USA). For single transfection, 1 μg of plasmid was used per 1 × 10^5^ cells. For co-transfection with both shSmad3 and pSmad6 in the experimental arm or their respective vectors in the control arm, 800 ng of each plasmid was used per 1 × 10^5^ cells. Fibroblasts were cultured for 72 h after transfection before analyses, unless otherwise indicated. For co-treatment with VPA and/or TGF-β2, the transfected cells were allowed to recover for 24 h post-transfection before incubation with the indicated treatments for 72 h.

### Mouse model of GFS

All experiments with animals were approved by the Institutional Animal Care and Use Committee (IACUC) and treated in accordance with the Association for Research in Vision and Ophthalmology (ARVO) Statement on the Use of Animals in Ophthalmic and Vision Research. NIH3T3/BL6 mice were obtained from the National University of Singapore Centre for Animal Resources. Experimental surgery was performed as described previously [21] on one eye while the contralateral unoperated eye was used as baseline for comparison. VPA was injected at 300 μg/ml (5 μl) into the conjunctiva of the operated eye immediately after and on day 2 post-surgery if the tissues were harvested on day 7 post-surgery. PBS was used as control and given at the same regimen.

### Real-time quantitative PCR analysis

Conjunctival fibroblasts and conjunctival tissues were processed and analysed as described previously [22, 23]. All PCR reactions were performed in triplicate in each set of experiment and three sets of independent experiments were performed for every interrogation, unless otherwise indicated. All mRNA levels were measured as C_T_ threshold levels. The best housekeeping gene for each experimental condition was determined using the NormFinder software [24]. Values were calculated as fold change by the 2^−∆∆CT^ method. Data is presented as the mean of the fold changes derived from each of the three sets of independent experiments comparing the relevant conditions, unless otherwise indicated. Primers for 18S rRNA were described previously [23]. Other primer sequences were as follows: Acta2-forward, 5′-CTGCCGAGCGTGAGATTG-3′ and Acta2-reverse, 5′-ATAGGTGGTTTCGTGGATGC-3′; Actb-forward, 5′-CACCCGCGAGCACAGCTTCT-3′, and Actb-reverse, 5′-CGTTGTCGACGACCAGCGCA-3′; Gapdh-forward, 5′- GCCAAGGCTGTGGGCAAGGT-3′, and Gapdh-reverse, 5′-TCTCCAGGCGGCACGTCAGA-3′; Rpl13a-forward, 5′-GAGGTCGGGTGGAAGTACCA-3′, and Rpl13a-reverse, 5′-TGCATCTTGGCCTTTTCCTT-3′; Col1a1-forward, 5′-CCCACCCCAGCCGCAAAGAG-3′, and Col1a1-reverse, 5′- GCCATGCGTCAGGAGGGCAG-3′; Smad2-forward, 5′- ATGTCGTCCATCTTGCCATTC-3′, and Smad2-reverse, 5′-AACCGTCCTGTTTTCTTTAGCTT-3′; Smad3-forward, 5′- CATTACCATCCCCAGGTCAC-3′, and Smad3-reverse, 5′-CGTAATTCATGGTGGCTGTG-3′; Smad4-forward, 5′-GTTCAGGTAGGAGAGACGTTTAAGGT -3′, and Smad4-reverse, 5′- CCTTTACATTCCAACTGCACTCCT-3′; Smad6-forward, 5′-TACCACTTCAGCCGGCTCTG-3′, and Smad6-reverse, 5′-AGTACGCCACGCTGCACCAGT-3′; Smad7-forward, 5′- GACTCCAGGACGCTGTTGGT-3′, and Smad7-reverse, 5′-CCATGGTTGCTGCATGAACT-3′; Tnc-forward, 5′-ACCATGCTGAGATAGATGTTCCAAA-3′, and Tnc-reverse, 5′-CTTGACAGCAGAAACACCAATCC-3′.

### Real-time cell proliferation analysis

The xCelligence real-time cell analyser (Roche Diagnostics GmbH, Penzberg, Germany) was used to assess cell proliferation according to manufacturer’s instructions. Fibroblasts were seeded onto the E-Plate 96 (Roche Diagnostics GmbH) wells at 2000 cells/well in normal culture medium, in quadruplicates. Adherent cell growth/density was monitored continuously for up to 5 days.

### Annexin V and viability assays by flow cytometry

Apoptosis was measured using the Guava Nexin Reagent (Guava Technologies, Hayward, CA, USA) while cell viability was measured using the Guava ViaCount assay (Guava Technologies) by flow cytometry. Primary mouse conjunctival fibroblasts were trypsinized at 72 h post-VPA treatment and processed according to manufacturer’s instructions. Each condition was performed in triplicates. Five thousand cells were analysed for each sample. Cell populations were quantified using the Guava EasyCyte Plus flow cytometry system (Guava Technologies).

### Immunoblotting

Whole cell lysates and tissue lysates were resolved by SDS-polyacrylamide gel electrophoresis followed by immunoblotting as previously described [22, 23]. Anti-type I collagen was from MD Bioproducts (St Paul, MN). Antibodies against SMAD2/3 and SMAD6 were from Cell Signaling Technology (Danvers, MA) and Abcam Plc (Cambridge, UK), respectively. The Smad2 (pSer465/467) antibody was obtained from Calbiochem (EMD Millipore, San Diego, CA). Both antibodies against SMAD4 and GAPDH were from Santa Cruz Biotechnology, Inc. (Santa Cruz, CA). Horseradish peroxidase (HRP)-conjugated secondary antibodies were from Jackson Immunoresearch Laboratories, Inc. (West Grove, PA). Densitometric analyses, where potential errors in loading were corrected to levels of the housekeeping GAPDH, were performed as reported previously [22]. Three sets of independent experiments were performed for every interrogation, unless otherwise indicated. Densitometric data is presented as the mean of the fold changes derived from each of the three sets of independent experiments comparing the relevant conditions, unless otherwise indicated.

### Immunofluorescence and picrosirius red polarisation microscopy

Cryosections of day 7 post-operated eye tissues and picrosirius red staining were performed as described previously [21]. Type I collagen antibody was obtained from MD Bioproducts (St. Paul, MN). Labeling by the primary antibodies was detected using secondary antibodies conjugated to Alexa Fluor-594 (Invitrogen, Eugene, OR). Nuclei were visualised by mounting the cells in DAPI-containing Vectashield mounting medium (Vector Laboratories, CA, USA). Labeled cells were visualised using the Zeiss Imager.Z1 microscope (Carl Zeiss Inc., USA) while polarisation microscopy was performed using the Nikon Eclipse Ti microscope (Nikon Instruments Inc., NY, USA).

### Reporter gene assays

The mouse ColI 3-0 Basic reporter construct includes 3200 nt of mouse collagen α1(I) promoter cloned into the pGL3-Basic vector [25]. The p3TP-lux reporter plasmid, a chimeric TGF-β-inducible reporter comprising of three multimerized TGF-β-binding elements, was a kind gift from Dr. Joan Massague (Memorial Sloan-Kettering Cancer Center, New York). 0.5 μg of reporter or control constructs were co-transfected into 1 × 10^5^ fibroblasts with 0.5 μg of the internal control pRL-TK (Promega) using the P2 primary cell 4D Nucleofector kit L (Lonza, Basel, Switzerland) and the 4D-Nucleofactor X unit (Lonza) according to manufacturer’s protocol. The transfected cells were then treated with VPA and/or TGF-β2 for 24 h the following day. Luciferase activity was measured via the Tecan Infinite M200 reader (Tecan Trading AG, Switzerland) using the Dual-Glo® Luciferase Assay system (Promega) according to the manufacturer’s protocol and values were normalised to Renilla luciferase activity. Each set of experiment was performed in triplicate and three sets of independent experiments were performed for each reporter assay. Data is presented as the mean of the fold changes derived from each of the three sets of independent experiments comparing the relevant conditions, unless otherwise indicated.

### Statistical analysis

Data are expressed as mean ± standard deviation (SD). Where only two treatment conditions were compared, the significance of differences between the two conditions was determined by the two-tailed Student’s *t* test using the Microsoft Excel 5.0 software, with significance at *p* < 0.05. Where more than two treatment conditions were compared, the significance of differences between the conditions was determined by one-way ANOVA using SPSS statistics. Bonferroni post hoc adjustment was applied to determine which conditions were significantly different from each other.

## Results

### VPA inhibits steady-state type I collagen expression in primary conjunctival fibroblasts

**Fig. 1:**
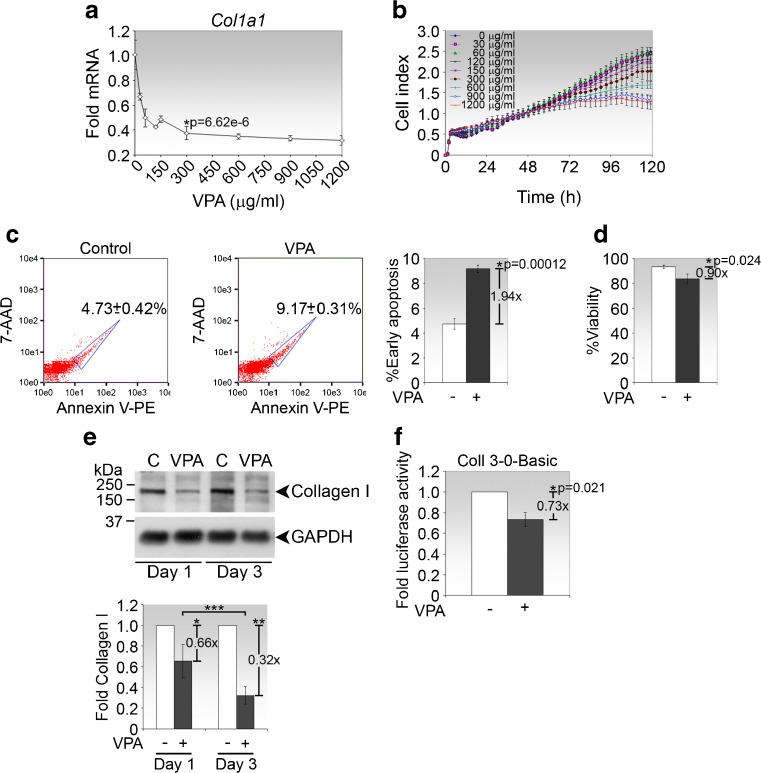
VPA inhibits steady-state type I collagen expression. **a** Real-time PCR analysis of *Col1a1* expression in primary conjunctival fibroblasts treated with increasing concentration of VPA for 72 h. Data are presented as mean fold change ± SD relative to untreated cells and are representative of three independent sets of experiments. *p value comparing fold mRNA between treatment with 300 μg/ml VPA and untreated controls is shown. **b** Real-time cell proliferation analysis of fibroblasts treated with 300 μg/ml VPA. Data are presented as mean cell index ± SD of triplicates. **c** Early apoptosis of conjunctival fibroblasts treated with VPA for 72 h. *Left and middle panels*, scatter plots of representative control and VPA-treated samples with gatings and mean percentages of early apoptotic cells indicated; *right panel*, data are presented as % early apoptotic cells ± SD of triplicates. *p value and the fold increase in early apoptotic cells in VPA-treated cells compared to controls are shown. **d** Viability of conjunctival fibroblasts treated with VPA for 72 h. Data are presented as % viable cells ± SD of triplicates. *p value and the fold change in cell viability in VPA-treated cells compared to controls are shown. **e** Immunoblot analysis of type I collagen in fibroblasts treated with VPA for the indicated times. Representative immunoblots from one experiment are shown. Densitometry values, normalised to GAPDH, represent the means ± SD of the folds expression of type I collagen in VPA-treated relative to untreated cells. *p* values comparing VPA treatment to control for each time point are *p = 0.00145 and **p = 0.0000315. *p* value comparing fold changes between day 1 and day 3 is ***p = 0.00173. C, control untreated cells. **f** Basal *Col1a1* promoter activity analysis. Fibroblasts were transfected with the Coll 3-0-Basic reporter plasmid and treated with VPA for 24 h before firefly luciferase activity was measured. Data were normalised to Renilla luciferase activity from co-transfected pRL-TK and presented as mean fold luciferase activity ± SD of VPA-treated relative to untreated cells

To determine the optimum dose of VPA for inhibition of type I collagen expression, we performed a titration study of VPA on primary cultures of conjunctival fibroblasts. VPA at a concentration of 300 μg/ml (or 2 mM) was able to significantly reduce *Col1a1* mRNA expression (Fig. 1a) while causing growth retardation with an average 6 % loss in cell density compared to untreated cells over a growth period of 5 days (Fig. 1b). We further ascertained that VPA treatment for 72 h resulted in 4.4 % more early apoptotic cells (Fig. 1c) and 9.5 % less viable cells (Fig. 1d) compared to untreated cells cultured for the same length of time. Hence, VPA treatment of primary conjunctival fibroblasts at 300 μg/ml for 72 h is associated with lower cellular viability and higher apoptosis rate.

We further verified that VPA inhibited type I collagen expression at the protein level (Fig. 1e). Notably, extended exposure to VPA for 3 days compared to 1 day resulted in significantly greater suppression of type I collagen expression. To demonstrate that VPA regulates *Col1a1* at the transcriptional level, conjunctival fibroblasts were transfected with a reporter plasmid driven by the *Col1a1* promoter followed by treatment with 300 μg/ml VPA. We observed that VPA significantly reduced steady-state *Col1a1* promoter activity (Fig. 1f), confirming that VPA has the capacity to suppress basal *Col1a1* expression.

Since treatment with VPA at 300 μg/ml for 72 h was determined to be effective in significantly suppressing *Col1a1* expression with no greater than 10 % loss in cell viability, subsequent investigation of VPA effects were measured under these conditions, unless otherwise specified.

### VPA suppresses steady-state Smad2, Smad3 and Smad4 but induces Smad6 expression

**Fig. 2:**
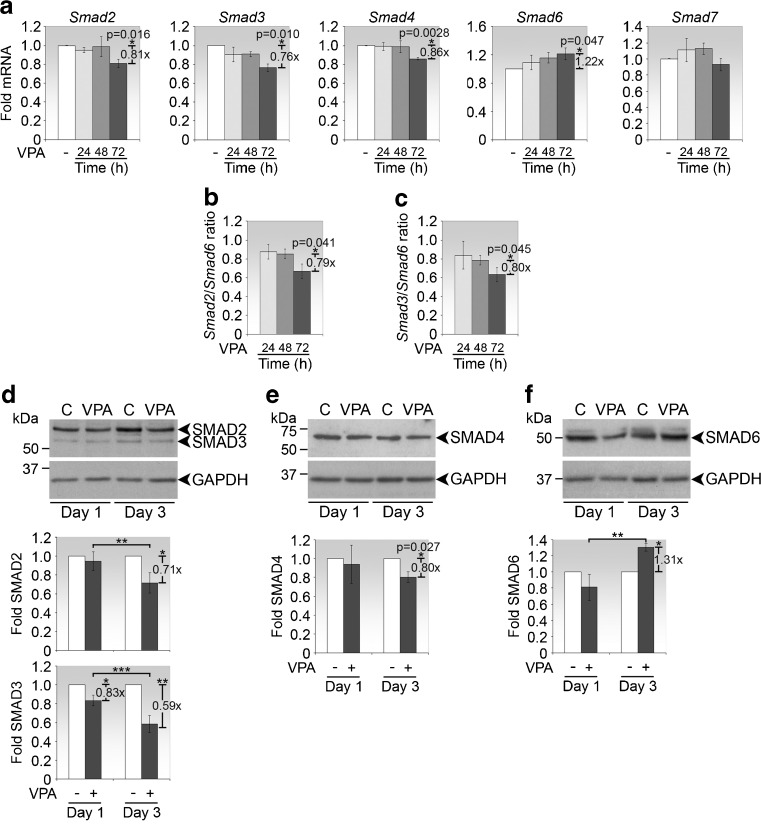
VPA exerts differential regulation on Smad family members. **a** Real-time PCR analyses of Smads in primary conjunctival fibroblasts treated with VPA for 24, 48 and 72 h. Data are presented as mean fold change ± SD relative to untreated control. *p values comparing fold mRNA between treatment with 300 μg/ml VPA for 72 h and untreated controls are shown. **b** Ratio of Smad2:Smad6 mRNA levels at 24, 48 and 72 h. The fold change and *p* value between 48 and 72 h are indicated. **c** Ratio of Smad3:Smad6 mRNA levels at 24, 48 and 72 h. The fold change and *p* value between 48 and 72 h are indicated. Immunoblot analyses of **d** SMAD2 and SMAD3, **e** SMAD4 and **f** SMAD6 in fibroblasts exposed to VPA for the indicated times. Representative immunoblots from one experiment of each SMAD are shown. *C,* untreated controls. Densitometry values, normalised to GAPDH, represent the means ± SD of the folds expression in VPA-treated relative to untreated cells of three (**e**, **f**), or four (**d**) independent sets of experiments. Fold changes and the associated *p* values, where significant, comparing the indicated conditions are shown. **d** SMAD2, *p = 0.00276 and **p = 0.011; SMAD3, *p = 0.013, **p = 0.000168 and ***p = 0.000888. **f** SMAD6, *p = 0.0237 and **p = 0.00218

Since Smads are implicated in the regulation of collagen expression, we examined the effect of VPA on Smads that are involved in the fibrogenic pathway. The influence of VPA on Smad transcript expression increased with increasing exposure time (Fig. 2a). VPA treatment for 72 h caused a significant downregulation of Smad2, Smad3 and Smad4 mRNAs by 19, 24 and 14 %, respectively, while Smad6 transcripts were upregulated by 22 % (Fig. 2a). VPA had no significant effect on Smad7 mRNA expression. The ratio of the mRNA levels of positive Smad2 and negative Smad6 mediators of SMAD signaling (Smad2 mRNA: Smad6 mRNA ratio) was decreased upon VPA treatment, and the difference was statistically significant between 48 and 72 h (Fig. 2b). A similar phenomenon was observed for the ratio of Smad3 and Smad6 mRNA levels (Fig. 2c). Hence, an exposure time of 72 h to VPA was sufficient to produce significant changes in Smad expression.

At the protein level, VPA also significantly inhibited SMAD2 and SMAD3 expression (Fig. 2d). Extended exposure to VPA for 3 days compared to 1 day also resulted in significantly greater suppression of SMAD2 and SMAD3 expression in a profile similar to that of type I collagen. Between the two, SMAD3 was reduced to a greater extent than SMAD2 at both time points. SMAD4 protein expression was also reduced with VPA treatment after 3 days (Fig. 2e). The profile of SMAD6 was the opposite, with significantly more SMAD6 being expressed after 3 days of VPA treatment (Fig. 2f). These data suggests a positive correlation between type I collagen expression with that of SMAD2, SMAD3 and SMAD4 and an inverse correlation with SMAD6.

### Smad3 and Smad6 contribute to steady-state type I collagen expression

**Fig. 3:**
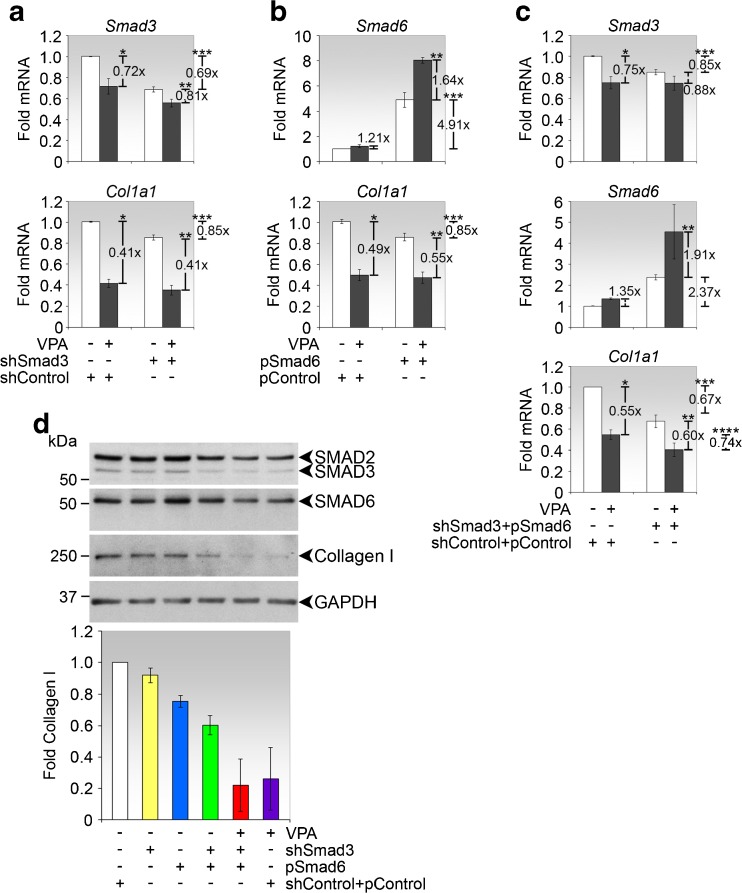
Smad3 and Smad6 contribute to the inhibitory activity of VPA on *Col1a1* expression. **a** Real-time PCR analyses of *Smad3* and *Col1a1* in conjunctival fibroblasts transfected with shSmad3 and/or treated with VPA, as indicated. *p* values comparing fold changes in *Smad3* expression between the indicated conditions are *p = 0.000258, **p = 0.0359 and ***p = 0.000130. *p* values comparing fold changes in *Col1a1* expression between the indicated conditions are *p = 9.889e−8, **p = 3.497e−7 and ***p = 0.00257. **b** Real-time PCR analyses of *Smad6* and *Col1a1* in conjunctival fibroblasts transfected with pSmad6 and/or treated with VPA, as indicated. *p* values comparing fold changes in *Smad6* expression between the indicated conditions are **p = 2.582e−8 and ***p = 2.575e−. *p* values comparing fold changes in *Col1a1* expression between the indicated conditions are *p = 2.735e−6, **p = 2.372−-5 and ***p = 0.0163. **c** Real-time PCR analyses of *Smad3*, *Smad6* and *Col1a1* in conjunctival fibroblasts co-transfected with shSmad3 and pSmad6 and/or treated with VPA, as indicated. *p* values comparing fold changes in *Smad3* expression between the indicated conditions are *p = 0.000887 and ***p = 0.0218. p value comparing fold changes in *Smad6* expression between the indicated conditions is **p = 0.0218. *p* values comparing fold changes in *Col1a1* expression between the indicated conditions are *p = 0.000193, **p=0.000820, ***p = 0.000226 and ****p = 0.0418. **a**–**c** Data are presented as mean fold change ± SD. **d** Immunoblot analyses of SMAD2, SMAD3 and SMAD6 in fibroblasts cultured under the indicated conditions. Representative immunoblots from one experiment are shown. Densitometry values, normalised to GAPDH, represent the means ± SD of the folds expression of type I collagen relative to control cells co-transfected with empty plasmid vectors

To determine in greater detail the involvement of Smad3 and Smad6 on VPA regulation of steady-state type I collagen expression, we used silencing and overexpression vectors of Smads to mimic the effects of VPA. Since VPA suppressed Smad3 expression, we simulated this effect in fibroblasts by transfecting them with small hairpin RNA of Smad3 (shSmad3). This method resulted in the significant reduction of Smad3 mRNA which was associated with a reduction in *Col1a1* mRNA, supporting the involvement of Smad3 in regulating steady-state *Col1a1* expression (Fig. 3a). Smad6 expression was not significantly affected by Smad3 downregulation (data not shown).

Conversely, since VPA induced Smad6 expression, we mimicked this effect in fibroblasts by transfecting them with a plasmid construct that overexpressed Smad6. This method resulted in an increase in Smad6 mRNA and an associated reduction in *Col1a1* mRNA without affecting Smad3 expression (data not shown), supporting the involvement of Smad6 in regulating steady-state *Col1a1* expression (Fig. 3b). Furthermore, the addition of VPA resulted in a greater increase in Smad6 transcripts, confirming the inductive effect of VPA on Smad6 expression.

We performed a further experiment to investigate the effect on *Col1a1* expression when fibroblasts were simultaneously transfected with shSmad3 and Smad6 overexpression vectors. This method resulted in greater reduction in *Col1a1* mRNA expression compared to transfection with either plasmid alone (Fig. 3c), corroborating the above data that both Smad3 and Smad6 are involved in regulating *Col1a1* expression. Moreover, greater *Col1a1* transcript reductions were observed when comparing plasmid transfections combined with VPA treatment against either VPA treatment or plasmid transfections alone (Fig. 3c), reinforcing the notion that *Col1a1* is regulable by all three factors.

We next performed immunoblotting experiments to verify that the inhibition of type I collagen by Smad3 downregulation, Smad6 overexpression and co-treatments with VPA were also reflected at the protein level (Fig. 3d). Indeed, exogenous regulation of Smad3 and Smad6 expression levels modulated type I collagen protein expression, with VPA being conspicuously the most effective in doing so. Taken together, our data indicate that basal *Col1a1* reduction by VPA is mediated in part by downregulating Smad3 and upregulating Smad6.

### VPA suppresses type I collagen induced by TGF-β2

**Fig. 4:**
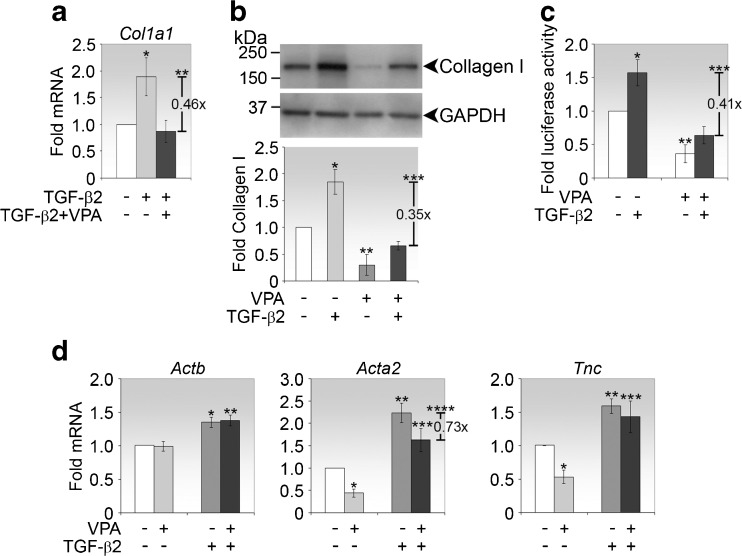
VPA suppresses the induction of type I collagen by TGF-β2. **a** Real-time PCR analyses of *Col1a1* in fibroblasts stimulated as indicated for 72 h. *p* value comparing fold changes in expression between TGF-β2-treated and untreated control cells is *p = 0.0113. *p* value comparing fold changes in expression between co-treatment (TGF-β2 + VPA) and TGF-β2 treatment is **p = 0.00561. **b** Immunoblot analyses of type I collagen in fibroblasts treated as indicated for 72 h. Representative immunoblots from one experiment are shown. Densitometry values, normalised to GAPDH, represent the means ± SD of the folds expression of type I collagen relative to untreated control. *p* values comparing fold changes in type I collagen expression between the indicated conditions and untreated control cells are *p = 0.000950 and **p = 0.00358. *p* value comparing fold changes in expression between co-treatment (TGF-β2 + VPA) and TGF-β2 treatment is ***p = 0.0000846. **c** TGF-β2-responsive promoter activity analysis. Fibroblasts were transfected with the p3TP-lux reporter plasmid and treated as indicated for 24 h before firefly luciferase activity was measured. Data were normalised to Renilla luciferase activity from co-transfected pRL-TK and represented as mean fold luciferase activity ± SD relative to untreated cells. *p* values comparing fold changes in luciferase activity between the indicated treatments and untreated cells are *p = 0.00559 and **p = 0.00259. *p* value comparing fold changes in luciferase activity between co-treatment (TGF-β2 + VPA) and TGF-β2 treatment is ***p = 0.000191. **d** Real-time PCR analyses of *Actb*, *Acta2* and *Tnc* in fibroblasts stimulated as indicated for 72 h. *p* values comparing fold changes in expression between treated and untreated control cells are *Actb* *p = 0.00120, **p = 0.000654; *Acta2* *p = 0.0267, **p = 0.000162, ***p = 0.0153 and ****p = 0.0173 (comparing between co-treatment (TGF-β2 + VPA) and TGF-β2 treatment); *Tnc* *p = 0.0202, **p = 0.00519 and ***p = 0.0334

Since VPA modulates Smad expression levels, it is reasonable to predict that VPA would disrupt TGF-β signaling. We therefore investigated the effect of VPA on the induction of *Col1a1* by TGF-β2. As expected, TGF-β2 alone significantly induced type I collagen expression at both mRNA (Fig. 4a) and protein levels (Fig. 4b). However, in the presence of VPA, the influence of TGF-β2 on type I collagen expression was subdued at both transcript (Fig. 4a) and protein levels (Fig. 4b), indicating that the ability of TGF-β2 to induce collagen above control levels is regulable by VPA.

It is possible that the failure of TGF-β2 to induce collagen to above control levels may be due to either lower baseline collagen levels caused by VPA and/or the inhibition of TGF-β2 signaling by VPA. To address the second possibility, we tested VPA in a reporter assay using the p3TP-Lux vector, a widely used artificial promoter construct designed to demonstrate maximal responsiveness to TGF-β [26] which can also be activated by Smad3 and Smad4 overexpression [27]. Conjunctival fibroblasts were transfected with the p3TP-Lux reporter plasmid followed by treatment with VPA and/or TGF-β2 for 24 h. We observed that VPA significantly reduced steady-state as well as TGF-β2-induced promoter activity of the p3TP-Lux transcriptional reporter (Fig. 4c). Markedly, type I collagen expression (Fig. 4b) and p3TP-Lux activity (Fig. 4c) appeared to share a similar response profile under the indicated conditions. Taken together, these data reveal that VPA has the capacity to modulate the activity of the p3TP-Lux promoter with or without TGF-β2, possibly contributed in part by reduced Smad3 which we have shown to be significantly reduced by 24 h (Fig. 2d). These observations also confirm that collagen expression is sensitive to both TGF-β2 and VPA activities.

Since TGF-β is known to induce myofibroblast transformation in fibroblasts, we also examined the impact of VPA on this activity. In conjunctival fibroblasts, three markers for myofibroblast differentiation, β-actin (*Actb*), α-smooth muscle actin (*Acta2*) and tenascin C (*Tnc*), were induced by TGF-β2 (Fig. 4d). Of these three genes, VPA modulated the basal mRNA levels of *Acta2* and *Tnc2*, but not *Actb*. However, unlike *Col1a1*, the mRNAs of *Acta2* and *Tnc2* remained above control levels when simultaneously treated with TGF-β2 and VPA. We therefore assume that myofibroblast differentiation induced by TGF-β2 may not be blocked by VPA under the present experimental conditions. This finding implies that the pathways regulating collagen induction and the myofibroblast phenotype acquired by TGF-β2 stimulation are multiple and overlapping and subject to differential sensitivities to VPA regulation in conjunctival fibroblasts.

### VPA abrogates the suppression of Smad6 by TGF-β2

**Fig. 5:**
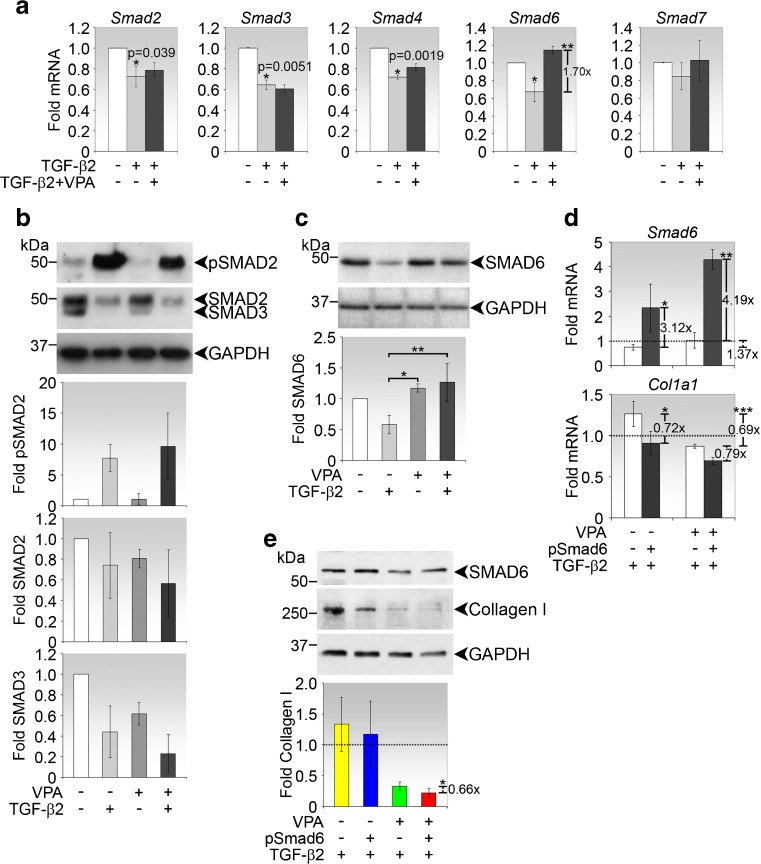
VPA modulates TGF-β2 effects on *Col1a1* and *Smad6* expression. **a** Real-time PCR analyses of Smad expression in fibroblasts stimulated as indicated for 72 h. Data are presented as mean fold change ± SD relative to untreated cells. *p values comparing fold changes in expression between TGF-β2-treated and untreated control cells are shown where significant. *p* value comparing fold changes in expression between co-treatment (TGF-β2 + VPA) and TGF-β2 treatment is **p = 0.0074. **b** Immunoblot analyses of phosphorylated SMAD2 (pSMAD2), SMAD2 and SMAD3 in fibroblasts treated as indicated for 72 h. Representative immunoblots from one experiment are shown. Densitometry values, normalised to GAPDH, represent the means ± SD of the folds expression relative to untreated control. **c** Immunoblot analyses of SMAD6 in fibroblasts treated as indicated for 72 h. Representative immunoblots from one experiment are shown. Densitometric analyses were performed as before. *p* value comparing fold SMAD6 expression between the indicated conditions are *p = 0.0199 and **p = 0.00849. **d** Real-time PCR analyses of *Smad6* and *Col1a1* in conjunctival fibroblasts transfected with pSmad6 and/or treated with VPA in the presence of TGF-β2. Data are presented as mean fold change ± SD relative to cells transfected with control plasmid, as represented by the *dotted line. p* values comparing fold changes in *Smad6* expression between the indicated conditions are *p=0.0461 and **p=0.000511. *p* values comparing fold changes in *Col1a1* expression between the indicated conditions are *p = 0.0232 and ***p = 0.0135. **e** Immunoblot analyses of SMAD6 and type I collagen in fibroblasts subjected to the indicated conditions. Representative immunoblots from one experiment are shown. Densitometry values represent the mean fold expression ± SD of type I collagen relative to cells transfected with control plasmid, as represented by the *dotted line. p* value comparing fold changes in type I collagen expression between co-treatment (TGF-β2 + VPA) and co-treatment cum SMAD6 overexpression is *p = 0.0043

We next determined whether VPA has the capacity to modulate TGF-β2 regulation of Smad expression. TGF-β2 alone significantly suppressed the transcript expression of Smad2, Smad3, Smad4 and Smad6 mRNAs in fibroblasts upon stimulation for 72 h (Fig. 5a). The reduction of SMAD2 and SMAD3 upon TGF-β2 stimulation was similarly observed at the protein level, with SMAD3 being affected by a greater extent compared to SMAD2 (Fig. 5b). Moreover, VPA did not impede the capacity of TGF-β2 to phosphorylate SMAD2 (Fig. 5b).

While co-treatment with TGF-β2 and VPA did not cause further repression of Smad2, Smad3 and Smad4 transcripts (Fig. 5a), the presence of VPA counteracted the inhibitory effect of TGF-β2 on Smad6 at both the mRNA and protein levels (Fig. 5a, c). To determine the involvement of Smad6 in the TGF-β2-induction of type I collagen expression, we stimulated the fibroblasts with TGF-β2 in combination with Smad6 overexpression. Overexpression of Smad6 can significantly reduce the induction of *Col1a1* transcripts by TGF-β2 (Fig. 5d). We further performed immunoblotting experiments to verify that the modulatory relationship between TGF-β2, Smad6 and VPA also occurs at the protein level. As can be observed, the interplay between SMAD6 overexpression and TGF-β2 stimulation resulted in variable induction of collagen from the independent primary cultures (Fig. 5e). However, the response of these cells to VPA was stable and highly reproducible. Furthermore, SMAD6 overexpression further enhanced the inhibitory effect of VPA on the induction of type I collagen by TGF-β2, confirming that SMAD6 is involved in the regulation of type I collagen expression.

### VPA inhibits type I collagen and upregulates Smad6 in the mouse model of GFS

**Fig. 6:**
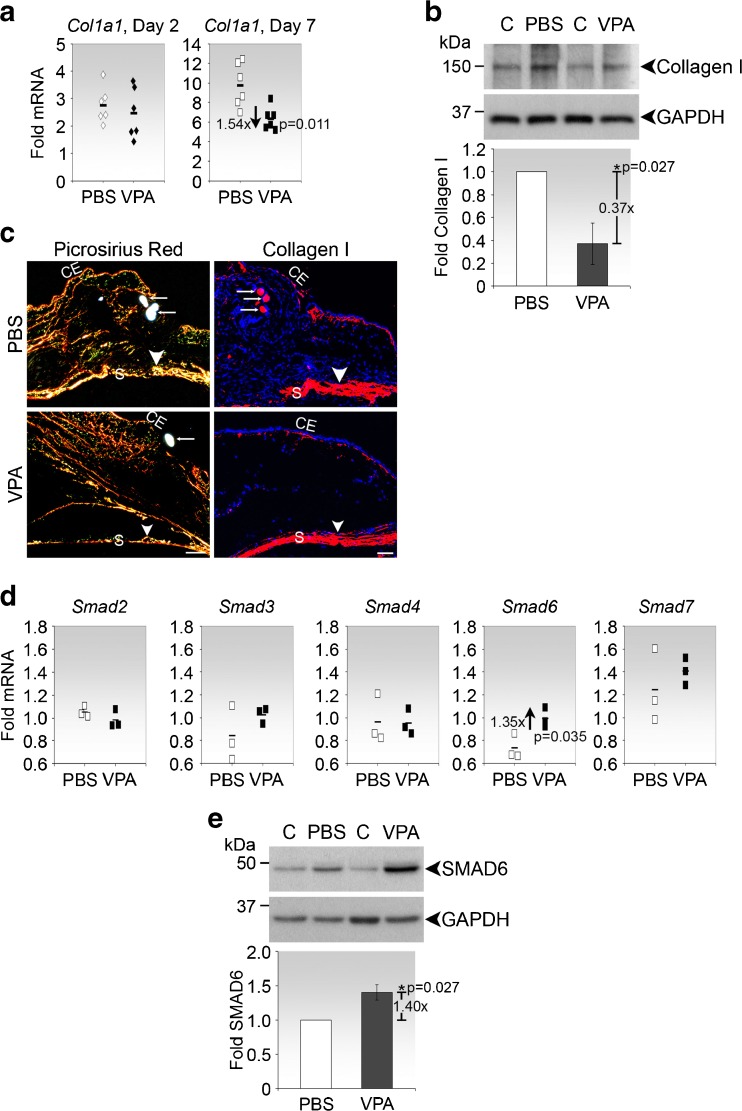
VPA suppresses type I collagen and upregulates Smad6 in the late phase of conjunctival wound healing. **a** Real-time PCR analyses of *Col1a1* expression in the day 2 and day 7 post-operative tissues. *Each symbol* represents a pool of 5 operated left eyes and is calculated as fold change over the corresponding pool of 5 contralateral un-operated right eyes. *Horizontal bars* represent the mean fold change for each experimental arm. The mean fold change and *p* value comparing VPA treatment relative to PBS control on day 7 are shown. **b** Immunoblot analyses of type I collagen expression in the day 7 operated conjunctival tissues. Representative immunoblots from one experiment are shown. Densitometry values, normalised to GAPDH, represent the means ± SD of the folds expression of type I collagen in VPA relative to PBS treated tissues from 3 independent experiments, each consisting of pooled tissues from 5 eyes of each treatment condition. The mean fold change and *p* value comparing the two conditions are shown. *C,* contralateral un-operated conjunctival tissues. **c** Histochemical and immunofluorescence analyses of collagen deposition in cryosections of the day 7 operated tissues. *Left panels*, cryosections stained with picrosirius red and visualised by polarised microscopy. *Scale bar*, 200 μm. *Right panels*, cryosections immunostained with anti-type I collagen antibodies (*red*). Nuclei were visualised by DAPI staining (*blue*). *Arrows* indicate sutures. *Scale bar*, 50 μm. *CE,* conjunctival epithelium; *S,* sclera. **d** Real-time PCR analyses of Smad genes in the day 7 operated conjunctival tissues. The meaning of each *symbol* is as described in (**a**). **e** Immunoblot analyses of SMAD6 in the day 7 operated conjunctival tissues. Representative immunoblots from one experiment are shown. Densitometric analysis, shown below the representative blot, was performed as stated in (**b**)

To verify that VPA exerts similar effects on type I collagen and Smad expression after experimental surgery on the conjunctiva, we injected the drug into the conjunctiva in the mouse model of GFS. We determined that VPA suppressed *Col1a1* transcript and protein expression in the late phase of wound healing (day 7 post-surgery) but not in the early phase (day 2 post-surgery) (Fig. 6a, b). Visualisation by polarised microscopy of picrosirius red-stained bleb cryosections as well as immunofluorescent analysis indicated a clear reduction in collagen fibres which was particularly conspicuous near the episclera of the VPA-treated day-7 surgical site compared to PBS control (Fig. 6c, arrowheads).

As with *Col1a1*, the injection of VPA did not cause significant differences in Smad expression in the early phase of wound healing (day 2 post-surgery) (data not shown). However, in the late phase of wound healing, Smad6 mRNA and protein levels were significantly and specifically upregulated in response to VPA treatment (Fig. 6d, e). Hence, VPA is also effective as a regulator of type I collagen in vivo and likely to involve Smad6 mediation. Moreover, similarities between the late-phase wound healing response, which likely involves TGF-β2 [23], and the response of conjunctival fibroblasts to VPA in the presence of TGF-β2 support the notion that these cells are key responders to VPA and TGF-β2 in the late phase of conjunctival wound healing.

## Discussion

To our knowledge, the present study is the first to demonstrate that VPA is an effective inhibitor of type I collagen expression in conjunctival fibroblasts in the steady-state as well as in the presence of the fibrogenic cytokine TGF-β2 via the selective regulation of Smads. The mechanism for collagen regulation in the steady-state by VPA involved the downregulation of Smad2, Smad3 and Smad4 and the upregulation of Smad6. On the other hand, VPA regulated TGF-β2-induced collagen expression selectively via Smad6. The influence of VPA on *Col1a1* and Smad6 was reiterated in the experimentally operated conjunctiva during the late phase of wound healing when TGF-β2 is likely to drive fibrotic development [23].

Our data is in agreement with previous studies which implicated Smad3 in the regulation of steady-state type I collagen expression. Overexpression of Smad3 stimulated basal *Col1a1* [28] and *Col1a2* [29] promoter activities, independent of TGF-β. Moreover, elevated Smad3 was observed in scleroderma fibroblasts which are characterised by the excessive synthesis and accumulation of matrix proteins [30]. Conversely, Smad3-deficient hepatic stellate cells expressed reduced *Col1a1* mRNA [5]. Similarly, we showed that suppression of Smad3 by either shRNA or VPA was able to reduce steady-state *Col1a1* expression.

Smad3 may regulate *Col1a1* expression by interaction with DNA in association with other transcription factors. Smad3 complexed with Smad4 is essential for the former’s DNA binding activity, independent of TGF-β [27, 31]. On the other hand, Smad2 complexed with Smad4 was able to bind DNA only in the presence of TGF-β [31], suggesting that it is unlikely for downregulation of Smad2 by VPA to have a significant impact on steady-state *Col1a1* expression. Incidentally, Smad4 was demonstrated to be necessary for basal type I collagen expression in kidney mesangial cells [32]. Hence, reduced Smad3 coupled with decreased expression of its partner Smad4 by VPA is likely to strengthen the suppression of *Col1a1* basal promoter activity ensuring reduced steady-state type I collagen expression. Moreover, Smad3 has been detected in a complex with AP-1, a key transcription factor in profibrogenic signaling [33]. Increased binding of the AP-1 complex to DNA via Smad3/Smad4 co-overexpression was shown to be sufficient for transcriptional activation [27]. Most importantly, this complex is likely to be important for collagen regulation since VPA disrupted the binding of the AP-1 complex to DNA in pancreatic stellate cells, resulting in reduced collagen synthesis [34]. Our data and observations made by others therefore suggest that VPA may reduce steady-state collagen expression on two fronts: by reducing Smad3/Smad4 levels as well as disrupting the interaction of the Smad3/Smad4/AP-1 complex with DNA.

Reduction of steady-state type I collagen expression by VPA is also likely to be exacerbated by increase in inhibitory Smad6 expression by VPA. Smad6 was reported to be associated with HDAC [35] which may have a role in promoting protein degradation, as has been shown for Smad7. Smad7 is known to be acetylated by p300/CBP which protects it from ubiquitination and TGF-β-induced degradation [36]. Smad7 interaction with HDACs promoted its degradation by deacetylation [37]. We therefore speculate that Smad6 may be similarly regulated and VPA may enhance Smad6 stability via HDAC inhibition.

The steady-state cellular localization of SMAD6 to both the nucleus and the cytosol [38, 39] suggests that Smad6 has the capacity to regulate basal Smad-mediated transcription activity. Smad6 was suggested to function as a transcription repressor or co-repressor by tightening the chromosome structure via complex formation with HDAC and binding to DNA [35]. Since VPA treatment was able to intensify *Col1a1* inhibition by Smad6 overexpression, the role of HDAC-associated chromatin-remodeling in *Col1a1* gene transcription regulation is likely not a predominant one. The issue of whether Smad6 regulates steady-state *Col1a1* expression by competing with Smad4 to form an inactive complex with R-Smads [40] remains to be investigated.

We have elected to investigate the interaction of VPA with TGF-β2 activities in conjunctival fibroblast collagen expression since this is the major isoform of the TGF-β family involved in subconjunctival fibrosis [41, 42]. Moreover, we have previously shown that TGF-β2 was induced in the late phase of wound healing in a mouse model of GFS and therefore likely to be a key source of the fibrogenic signal in vivo [23]. The relevance for modulating HDAC activity in the presence of TGF-β is underscored by the demonstration that TGF-β1 induced a general increase in HDAC activity in human osteoblasts and treatment with VPA was able to alter TGF-β1 signaling in these cells [43].

Numerous studies have implicated Smad3 as the key mediator of the fibrogenesis signaling pathway incited by TGF-β signals [4, 44]. However, our study revealed that modulation of TGF-β2 induction of collagen by VPA selectively involved Smad6. Parallels may be drawn between the impact of VPA and TGF-β2 on Smad6 expression and the capacity of trichostatin A, also an HDAC inhibitor, to rescue TGF-β1-suppressed Smad7 in nasal polyp-derived fibroblasts [45]. Our finding is unanticipated because Smad6 is best known as a specific inhibitor of BMP signaling [46] whereas Smad7 is commonly associated with TGF-β/activin signaling [47]. However, recent evidence indicated that Smad6, but not Smad7, interfered with non-canonical TGF-β signaling by negatively regulating the TRAF6-TAK1-p38 MAPK/JNK pathway [48]. Given that signaling pathways mediated by ERK1/2 MAPK, p38 MAPK and PI3K/Akt/PKB are known to be important in regulating collagen expression [1], collagen induction by TGF-β2 essentially involves both Smads and Smad-independent non-canonical TGF-β pathways. Indeed, the additive nature of Smad signaling and non-canonical pathways such as p38 MAPK signaling in TGF-β regulation of collagen expression has been reported [49]. Our study now brings to light the potential involvement of Smad6 in negatively regulating Smad-independent non-canonical TGF-β pathways that lead to collagen induction and fibrogenesis. It is therefore conceivable that targeting Smad6 will be beneficial as part of an anti-fibrogenic therapeutic strategy.

The differential effects of VPA on the induction of myofibroblast markers and collagen by TGF-β2, suggest that multiple pathways, with potential overlaps, may be involved in delivering these two end-points. Indeed, myofibroblast differentiation induced by TGF-β involves not only Smads [50] but other pathways such as integrin signaling [51]. The involvement of multiple overlapping pathways may explain why TGF-β2-induced *Col1a1* was effectively suppressed by VPA but not similarly induced *Acta2* and *Tnc*, although all three were highly regulable by VPA per sé. These observations serve to highlight the consistent effectiveness of VPA on collagen inhibition, be it in the steady-state or upon fibrogenic activation by TGF-β2.

Collectively, our data clearly implicated VPA as an effective therapeutic for reducing collagen accumulation in conjunctival fibrosis via a mechanism that involves Smads. On the other hand, our findings also allude to collagen deficiency as a potential side effect of systemic long-term use of VPA since it has the capacity to regulate steady-state collagen expression. Indeed, bone loss as a long-term side effect of VPA medication has been described [52]. Furthermore, a proteome study of a spinal muscular atrophy cell line treated with VPA revealed that out of more than one thousand proteins evaluated, collagens I and VI were strikingly reduced in these cells [53]. Hence, it is helpful that a eukaryotic receptor for VPA, which is homologous to the mammalian solute carrier family 4 (SLC4] bicarbonate transporter, has recently been identified [54]. Further in-depth evaluation of VPA cellular effects and mechanisms, coupled with regulated drug uptake, may help towards optimising the clinical benefits of VPA while minimising possible side effects.
